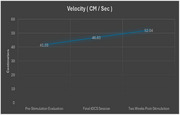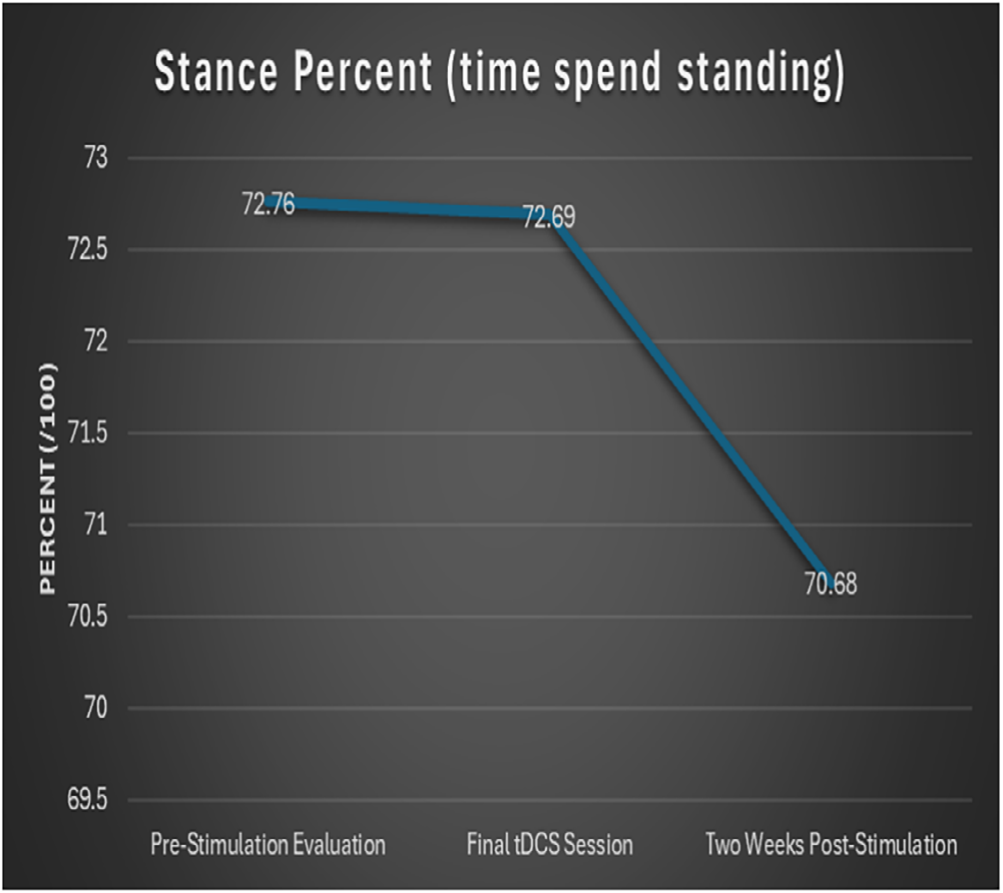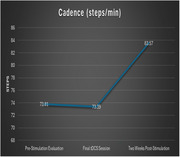# Daily Sessions of tDCS Improves Mobility in People with PSP and CBD

**DOI:** 10.1002/alz70858_105852

**Published:** 2025-12-26

**Authors:** Carlos Tyler Roncero, Anthony E. Lang, Durjoy Lahiri, Bruna Seixas Lima, Dvir Dori, Evan Dyke, Howard Chertkow

**Affiliations:** ^1^ Baycrest Academy for Research and Education, Toronto, ON, Canada; ^2^ Toronto Western Hospital, Toronto, ON, Canada; ^3^ Baycrest/Rotman Research Institute, Toronto, ON, Canada; ^4^ Rotman Research Institute‐Baycrest, TORONTO, ON, Canada; ^5^ Baycrest, Toronto, ON, Canada; ^6^ Baycrest and Rotman Research Institute, Toronto, ON, Canada

## Abstract

**Background:**

Many neurodegenerative diseases currently lack any effective form of therapy. Progressive Supranuclear Palsy (PSP) and Corticobasal degeneration (CBD) fall into this category. We hypothesized that transcranial direct current stimulation (tDCS) targeting the motor cortex and midbrain would improve walking capabilities when administered to people living with PSP and CBD during 20 minutes of walking. This hypothesis is supported by past literature suggesting the capacity of tDCS to increase cortical excitability, which in turn enhances motor performance.

**Method:**

10 participants (9 with PSP) completed 12 walking sessions over the course of three weeks, where week 1 consisted of SHAM stimulation (a placebo condition), while weeks 2 and 3 involved active tDCS with an anode electrode over Cz (the motor cortex) and a cathode electrode over the right deltoid muscle. Participants were blind to the stimulation received. Gait assessments at the end of week 1 (i.e., the end of the placebo week) were compared to results obtained at the end week 3 (i.e., the final day of stimulation), as well as two weeks after the final stimulation session, to check for any immediate and sustained gait improvements relative to the placebo (SHAM stimulation) phase. Three key gait parameters were examined: walking speed, rhythm, and stability, to quantify enhancements in speed, step cadence, and reduction in stop times as indicators of improved walking ability.

**Result:**

Despite the small sample size, we found that cadence and velocity were significantly improved two‐weeks post‐stimulation. More specifically, cadence (steps/min) improved from 73.81 at baseline to 83.57 two‐weeks later (*t*(9) = 3.05, *p* < .01), and velocity (cm/sec) improved from 41.55 at baseline to 52.04 two weeks later. Improvement was also found for stance percent, but the difference wasn’t significant.

**Conclusion:**

The significant improvements observed (10 to 20% improvement) encourage further investigation of tDCS for improving mobility in the management PSP and CBD. Neuromodulation is safe and a viable approach to improving symptoms in hitherto untreatable neurodegenerative diseases.